# Parent-Completed Developmental Screening in Premature Children: A Valid Tool for Follow-Up Programs

**DOI:** 10.1371/journal.pone.0020004

**Published:** 2011-05-26

**Authors:** Cyril Flamant, Bernard Branger, Sylvie Nguyen The Tich, Elise de La Rochebrochard, Christophe Savagner, Isabelle Berlie, Jean-Christophe Rozé

**Affiliations:** 1 Department of Neonatal Medicine, University Hospital, Nantes, France; 2 National Institute of Health and Medical Research CIC004, Nantes University Hospital, Nantes, France; 3 “Loire Infant Follow-up Team” (LIFT) Network, Pays de Loire, France; 4 Department of Neonatal Medicine, University Hospital, Angers, France; 5 Ined, Paris, France; 6 National Institute of Health and Medical Research, CESP, U1018, Le Kremlin-Bicêtre, France; 7 Université Paris-Sud, UMRS 1018, Le Kremlin-Bicêtre, France; University of Oxford, United Kingdom

## Abstract

Our goals were to (1) validate the parental Ages and Stages Questionnaires (ASQ) as a screening tool for psychomotor development among a cohort of ex-premature infants reaching 2 years, and (2) analyse the influence of parental socio-economic status and maternal education on the efficacy of the questionnaire. A regional population of 703 very preterm infants (<35 weeks gestational age) born between 2003 and 2006 were evaluated at 2 years by their parents who completed the ASQ, by a pediatric clinical examination, and by the revised Brunet Lezine psychometric test with establishment of a DQ score. Detailed information regarding parental socio-economic status was available for 419 infants. At 2 years corrected age, 630 infants (89.6%) had an optimal neuromotor examination. Overall ASQ scores for predicting a DQ score ≤85 produced an area under the receiver operator curve value of 0.85 (95% Confidence Interval:0.82–0.87). An ASQ cut-off score of ≤220 had optimal discriminatory power for identifying a DQ score ≤85 with a sensitivity of 0.85 (95%CI:0.75–0.91), a specificity of 0.72 (95%CI:0.69–0.75), a positive likelihood ratio of 3, and a negative likelihood ratio of 0.21. The median value for ASQ was not significantly associated with socio-economic level or maternal education. ASQ is an easy and reliable tool regardless of the socio-economic status of the family to predict normal neurologic outcome in ex-premature infants at 2 years of age. ASQ may be beneficial with a low-cost impact to some follow-up programs, and helps to establish a genuine sense of parental involvement.

## Introduction

Developmental outcome of preterm infants is a worthwhile concern for clinicians and research teams. Early detection of non-optimal neurodevelopment is essential for timely intervention in order to correct or attenuate problems. Standardized tests such as the Bayley scale, or in France the revised Brunet-Lezine scale [1], provide efficient measures of outcome. These tests performed by a specialised psychologist are time-consuming and not usable as a routine examination. Interest is growing in developing simpler, less expensive and time-consuming ways of ascertaining the development of children, such as using questionnaires for parents [2]. Studies have shown that most parents are able to correctly judge their children's performance, and that their concerns are appropriate [3]–[6]. Therefore, the production of a parent report cut-off score with good discriminatory power for the neurodevelopmental outcome of their child is of prime importance. Few parental questionnaires have shown significant agreement with standardized developmental test scores in children born preterm [7]–[10]. The Ages and Stages Questionnaires (ASQ) constitute a screening method of monitoring children who are at risk for developmental delay [11]. This structured questionnaire involving five domains of development has been shown to be cross-culturally valid between the United States and other Western settings [12]. To the best of our knowledge, only two studies of ex-premature infants have compared ASQ with a formal psychometric assessment [13], [14], and one study involved pediatric developmental impression [15]. No study has dealt with the association between ASQ and the revised Brunet-Lezine scale for extremely preterm children. Moreover, parental education and characteristics of parental socio-economic status influence measures of child development [16], and might affect the accuracy of parent reporting. Thus, mothers with a higher educational achievement and those who are not working may be more accurate in reporting their child's development [17]. A cohort of 703 ex-premature infants reaching 2 years gave us the opportunity to (i) validate the ASQ as a screening tool for abnormal development quotient in a French-speaking population, and (ii) analyse the influence of parental socio-economic status on the efficacy of the questionnaire.

## Methods

### Patients and data source

The study included all surviving children born between January 2003 and December 2006 at <35 weeks gestational age, and enrolled at the regional routine “Loire Infant Follow-up” Network, Pays de la Loire, France [18]. The “Loire Infant Follow-up Team” (LIFT) includes 24 maternity facilities, among which 3 are hospitals with neonatal intensive care units (Nantes, Angers, Le Mans). Written consent was obtained for each patient before their inclusion in the regional routine “Loire Infant Follow-up” Network. This network was registered to the French CNIL (Commission Nationale de l'Informatique et des Libertés) in order to gather data from clinical records.

### Neurodevelopmental assessment

Neurodevelopmental outcome at 2 years corrected age was assessed by clinical examination and a revised Brunet-Lezine test. Children reaching 2 years were first evaluated by trained pediatricians of our follow-up network. Children were classified as possessing optimal neuromotor development or non-optimal neuromotor development. Non-optimal neuromotor function was assigned when children were unable to walk without aid (cerebral palsy) or when the clinical examination revealed abnormal neurological signs (phasic stretch in the triceps surae muscle and imbalance of passive axial tone with predominance of extensor tone) during independent walking by a corrected age of 2 years [19].

The neurological outcome at the age of 2 years was also assessed by a specialized psychologist using the revised Brunet-Lezine test with establishment of a DQ score. This early childhood psychomotor development test was developed in France from 1943 and revised between 1994 and 1996 on a sample of 1032 French children [20]. The development of the initial Brunet-Lezine test and its revision followed rigorous methods, including the evaluation of test-retest reliability and internal reliability, both of which were high. The minimum duration of the test is 30 minutes. It is intended to enable 4 developmental age subscores to be calculated for children who are aged 2 to 30 months. The revised Brunet-Lezine test covers 4 domains (movement and posture, language, socialization, coordination) and allows calculation of 4 subscores which, when combined, yield a global DQ score. DQ values ≤85 define neurodevelopmental impairment. Infants who were not able to perform a DQ test because their neurologic impairment was too severe were included in the subgroup “DQ ≤85 or DQ not realizable”. Pediatric psychologists were blind to parental socio-economic status and maternal education.

### Ages and Stages Questionnaires (ASQ)

The ASQ is an American series of 19 age-specific questionnaires at intervals for the age range 4 to 60 months with a third edition recently published (Squires J. & Bricker D. 2009). The second version with the French translation of the 24-month questionnaire was used in the present study. The ASQ requires about 15 minutes to complete.

The questionnaire consists of 30 developmental items to assess five domains of child development: communication, gross motor, fine motor, problem solving and personal-social. For each item, the parents indicate “yes” (10 points), “sometimes” (5 points) or “not yet” (0 points) to represent their child's ability to perform a task. Each domain score was obtained by the sum of the items, compared with established screening cut-off points, and was considered abnormal when the score was 2 SD below the mean [11]. The global ASQ was scored as abnormal if one domain failed. The total sum of the five scores was also calculated.

Parents were invited to participate in the study when their child had a 2 years corrected age, taking into account the fact that the questionnaire is valid for 1 month either side of the 24-month target age (ASQ time frame). Parents were asked to complete the ASQ before the medical assessment and the 24-month evaluation by a psychologist, so that their observation of their child's response did not influence their responses to the questionnaire.

### Socio-economic survey

A phone survey was conducted by one of the authors (ELR), who questioned the parents about their job and maternal level of education. Considering the size of the entire cohort, a subset of the population selected by randomization underwent an additional socio-economic survey. Two indexes were built and used for the analysis: socio-economic status and maternal education. Each was treated as a 2-level categorical variable. Taking into account the best status of one of the two parents, the socio-economic variable was evaluated according to the job, depending on a scale between blue-collar workers until white-collar workers. A mother's education level was considered high when school education was maintained for more than 2 years after a high school diploma.

### Statistical analyses

All analyses were performed with SPSS 15.0. Medians, means and SDs are reported for a continuous variable and frequencies for categorical variables. Sensitivity, specificity and likelihood ratio results are expressed with a 95% confidence interval (95%CI). These data were computed to assess how ASQ parental assessment could correctly identify infants with optimal neurodevelopmental outcome. A receiver-operating-characteristic (ROC) curve was constructed to search for the optimal ASQ cut-off value to predict the DQ ≤85 in our sample. The Fisher's exact test and unpaired Mann-Whitney *U* test were used to assess the possible influence of socio-economic level and maternal education on clinical neurodevelopmental outcome, DQ score and ASQ parent report. The level of statistical significance was p <.05 for all analyses using two-tailed comparisons.

## Results

**Figure 1:**
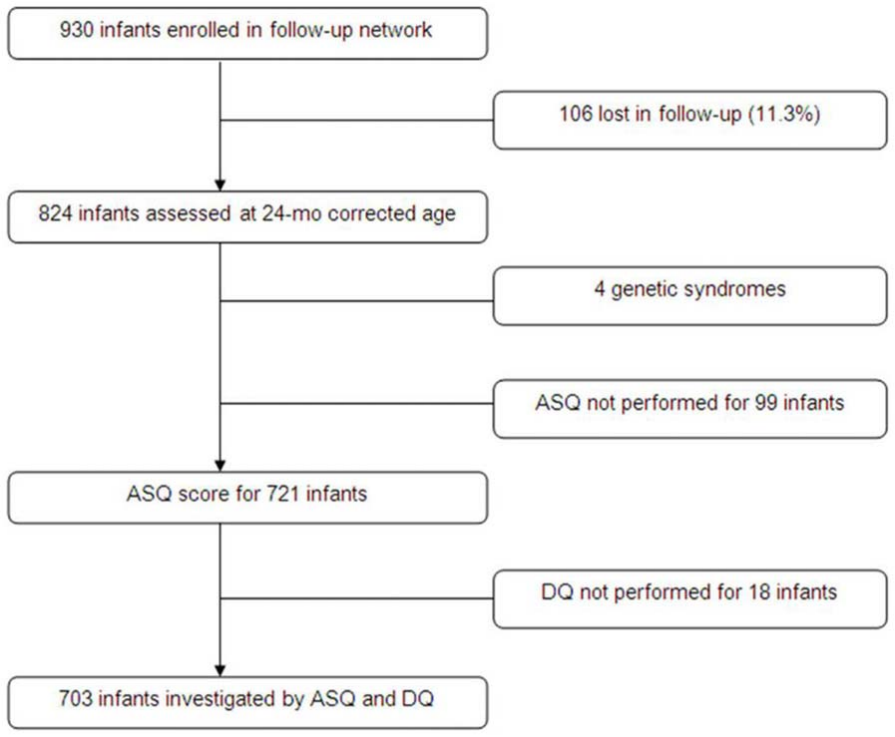
Cohort profile.

Eight-hundred and twenty-four infants of the 930 infants enrolled in the regional network attended a medical examination at 2 years of age (89%). As described in the cohort profile (Fig. 1), ASQ was assessed for 721 infants. Eighteen of these infants did not receive the Brunet Lezine test. At the end, 703 infants were included in the analysis. There were no significant differences between these 703 infants and the others (n = 227) with respect to gestational age (respectively 31.6 weeks GA ±2.3 vs. 31.8 weeks GA±2.3, p = 0.47) and birth weight (respectively 1662 gr ±530 vs. 1591 gr ±437, p = 0.33).

Detailed information regarding parental socio-economic status was available for 419 infants. The socio-economic level was scored as high for 219 infants, and low for 200 infants.

The characteristics of the study population are summarized in Table 1. At 24 months of corrected age, 630 infants (89.6%) had an optimal neuromotor outcome, and 73 (10.4%) were considered as having a non-optimal neuromotor outcome (29 with cerebral palsy and 44 with milder signs consistent with independent walking). The cognitive assessment was obtained for 673 infants. Thirty infants (4.3%), all with neurological impairment, were not able to take the test. The overall DQ ranged from 69.2 to 126.7 for a mean of 100.6±7.8 and a median of 101. DQ was ≤85 in 22 children (3.1%). Subscore analysis revealed that language was the most frequently abnormal score with 158 children impaired (22.5%). The classical ASQ classified 323 infants with 1 failed domain (46%) and 146 infants with 2 failed domains (21%). The most frequent failures were in the domains of communication (26%) and personal-social (18%). The global ASQ score ranged from 40 to 300 for a mean of 232.7±39.1 and a median value of 240.

**Table 1:** Characteristics of the population studied (n = 703).

**Infant**		
Gestational age (wk), median (range)	32	(30–34)
Birthweight (g), median (range)	1710	(1330–2040)
Male gender (%)	386	(54.9%)
Singleton (%)	522	(74.3%)
Neuromotor assessment (24-mo corrected age)		
Optimal, n (%)	630	(89.6%)
Non-optimal, n (%)	73	(10.4%)
**DQ assessment (24-mo corrected age)**		
DQ ≤85 or not realizable	52	(7.4%)
DQ ≤85	22	(3.1%)
DQ not realizable	30	(4.3%)
DQ, median (range)	101	(96–105)
Language score ≤85, n (%)	158	(22.5%)
Socialization score ≤85, n (%)	21	(3.0%)
Coordination score ≤85, n (%)	57	(8.1%)
Postural score ≤85, n (%)	24	(3.4%)
**ASQ assessment** * ** (24-mo corrected age)**		
Overall ASQ score, median (range)	240	(210–260)
Communication failed (%)	184	(26.2%)
Gross motor failed (%)	80	(11.4%)
Fine motor failed (%)	63	(9.0%)
Problem solving failed (%)	110	(15.6%)
Personal-social failed (%)	129	(18.3%)
1 domain failed (%)	323	(45.9%)
2 domains failed (%)	146	(20.8%)

DQ: Developmental Quotient (revised Brunet Lezine scale); ASQ: Ages and Stages Questionnaires;

*cut-off value for a positive screen is 2 SD below the mean on ASQ.

### ASQ and DQ correlations

**Figure 2:**
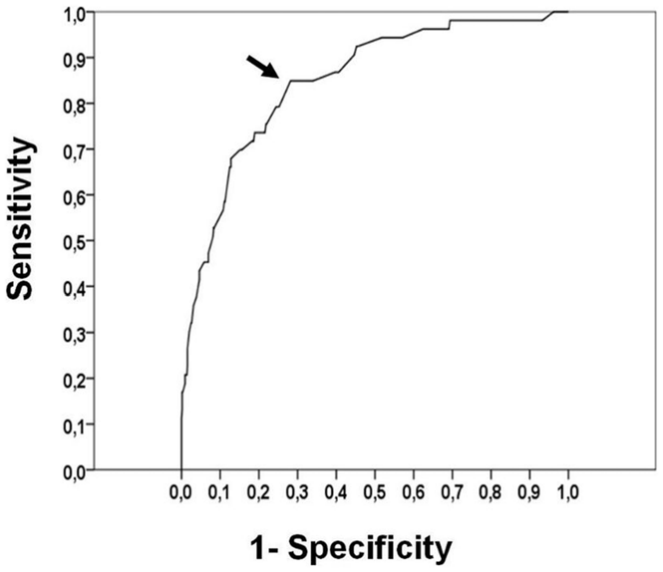
Receiver operating characteristic curve (ROC) for prediction of Development Quotient score ≤85 from ASQ. Arrow denotes optimal predictive value (ASQ score of 220).

When the ASQ was used as the standard (i.e. 1 domain failed on ASQ considered as a failed screen), the questionnaire had an optimal sensitivity of 0.88 (specificity of 0.57), whereas the sensitivity decreased to 0.60 using a definition of 2 ASQ domain failures (specificity of 0.82). Using the overall ASQ score as a continuous variable allowed us to build a receiver operator curve (ROC) in determining the DQ ≤85 (Fig. 2). ASQ scores produced an area under the ROC curve (AUC) value of 0.85 (95% confidence interval: 0.82–0.87). The optimal parent-report cut-off score for identifying a DQ ≤85 was an overall ASQ score of 220. Thus, a receiver operating characteristic-determined ASQ cut-off of ≤220 had optimal discriminatory power for identifying DQ ≤85 with a sensitivity of 0.85 (95%CI: 0.75–0.91) and specificity of 0.72 (95%CI: 0.69–0.75). The cross-tabulation of developmental classification using overall ASQ and DQ scores (Table 2) showed a positive likelihood ratio of 3 and negative likelihood ratio of 0.21 regarding the overall ASQ cut-off of 220. Amongst the subpopulation of 475 infants who scored higher than 220, only eight children had an abnormal DQ: 6 were not able to complete the test, and five of these were not able to perform the DQ language test even though the communication domain score of the ASQ was not failed. The 2 remaining infants had a DQ score of 84.1 and 84.9.

**Table 2:** Cross-tabulation of developmental classification using ASQ and DQ scores assessed by the revised Brunet-Lezine scale.

ASQ assessment	DQ ≤85 or not realizable [n = 52]	DQ >85[n = 651]	% Sensitivity(95%CI)	% Specificity(95%CI)	Positive likelihood ratio(95%CI)	Negative likelihood ratio(95%CI)
Overall ASQ ≤220 (%)	44	184	0.85 (0.75–0.91)	0.72 (0.69–0.75)	2.99 (2.60–3.45)	0.21 (0.13–0.37)
1 domain failed	46	277	0.88 (0.79–0.94)	0.57 (0.54–0.61)	2.08 (1.86–2.32)	0.21 (0.1–0.38)
2 domains failed	31	115	0.60 (0.46–0.73)	0.82 (0.80–0.85)	3.37 (2.67–4.26)	0.49 (0.37–0.65)

ASQ: Ages and Stages Questionnaires; DQ: Developmental Quotient; *% Sensitivity*, percent of “delayed” infants detected by the screening test as “delayed”; *% Specificity*, percent of “normally developing” infants detected by the screening test as “normal”.

### Socio-economic level, ASQ and outcome

In the 419 families whose socio-economic level was obtained, this index was clearly associated with neurodevelopmental outcome (Table 3). Optimal outcome was significantly more frequent among families with a high socio-economic level than those having a less favourable level (p = 0.02). Among infants with optimal neurodevelopmental outcome, the median value for the DQ score was associated with socio-economic level and maternal education (p = 0.001 and p = 0.008, respectively). However, in infants with non-optimal neurodevelopmental outcome, no correlation was found with socio-economic data. The median value for ASQ was not significantly associated with a family's socio-demographic characteristics, irrespective of the neurodevelopmental outcome group. Comparison of sensitivity and specificity of ASQ for prediction of a DQ score ≤85 revealed that this screening tool was not modified by socio-economic level or maternal education (see Table 3).

**Table 3:** Developmental quotient and ASQ score in relation to a family's socio-demographic characteristics.

24-mo infant's outcome	Socio-economic level		p	Maternal education: high school		p
	High (n = 219)	Low (n = 200)		Yes (n = 209)	No (n = 224)	
Optimal neurodevelopment, n (%)	194 (88.6)	159 (79.5)	0.02	183 (87.6)	182 (81.3)	0.10
DQ, median (interquartile)	103 (99–108)	100 (95–103)	0.001	103 (99–108)	100 (95–104)	0.008
ASQ, median (interquartile)	242 (225–261)	240 (220–260)	0.39	245 (225–260)	240 (220–260)	0.27
Non optimal neurodevelopment, n (%)	25 (11.4)	41 (20.5)	0.02	26 (12.4)	42 (18.8)	0.10
DQ, median (interquartile)	93 (85–103)	94 (84–100)	0.61	96 (85–104)	91 (84–101)	0.69
ASQ, median (interquartile)	200 (155–225)	200 (165–240)	0.49	203 (164–247)	200 (160–235)	0.57
ASQ ≤220 as predictor of DQ≤85						
Sensitivity (95%CI)	0.83 (0.60–0.94)	0.83 (0.65–0.93)	0.61	0.85 (0.58–0.96)	0.83 (0.61–0.94)	0.69
Specificity (95%CI)	0.74 (0.69–0.79)	0.69 (0.63–0.74)	0.36	0.76 (0.70–0.81)	0.69 (0.63–0.75)	0.17

ASQ: Ages and Stages Questionnaires; DQ: Developmental Quotient.

## Discussion

Our study of a large population-based cohort demonstrates that parental completion of ASQ is a simple, valid and cost-effective means of screening for normal neurodevelopmental outcome among ex-premature infants at 2 years of age. We also showed that, despite the influence of socio-demographic factors on neurodevelopmental outcome, the ASQ remains a valid tool regardless of the socio-economic status of the family.

We propose a new approach to the ASQ score by testing three different scoring systems. The classical score recommended by the University of Oregon's Center [11] (ASQ abnormal if one domain failed) first showed a good sensitivity of 0.88, but a rather low specificity of 0.57. Sices et al. related that certain clinicians use a broader definition of 2 failed domains on ASQ as a failed screen when scores are below, but near, the cut-off point [21]. We confirmed in our study that this definition considerably reduced the sensitivity of the tool to 0.6 (specificity of 0.82) as suggested by Sices et al. Our new approach for the overall ASQ score as a continuous variable, obtained by adding the scores of 5 domains, allowed us to determine a cut-off value of 220 as optimal for a good sensitivity (0.85) and specificity (0.72) in order to detect infants with a DQ ≤85. In a recent study, Marks et al. [15] showed the importance of lowering the threshold for administering a quality developmental screening instrument when providing surveillance for premature infants. In our study, in comparison with the ASQ classical score, an overall ASQ score enabled a reduction of preterm referral rates from 46% to 32%. With this new approach, 184 of the 703 infants would have been over-referred, and only 8 infants would have been missed. These infants had false negative results with an ASQ score >220, whereas they failed the DQ test. Two infants had a complete Brunet-Lezine scale with calculation of subscores covering 4 domains and a global DQ ≤85. On the other hand, the DQ test could not be completed for 6 infants. Five of these infants were not able to perform the DQ language domain test, whereas the communication domain score of the ASQ had not failed. It is possible that some children might be too shy to take part in this evaluation and that the language domain results in parent reports that are a particularly rich source of information concerning their child's emerging abilities, and may be more accurate than psychometric assessment.

Few studies of ex-premature infants have compared ASQ with formal psychometric assessment [13], [14]. In the study published by Skellern et al., ASQ was compared to different psychometric tests in an entire cohort of 136 infants born prematurely [13]. At the age of 24 months, the population was limited to 39 infants for which ASQ was significantly associated with the Griffith Mental Development Scale. The second study by Klamer et al. [14] showed a correlation between ASQ and the Wechsler Preschool and Primary Scales of Intelligence-Revised (WPPSI) among a population of 22 ex-preterm infants at the age of 35–44 months. A major strength of our study is that the data are established on a very large population-based cohort of preterm infants followed in our regional network. Considering the number of ASQ reports, our study strongly supports the validity of ASQ as a screening tool in comparison with the DQ test.

With a subgroup of 400 parents, we have the opportunity to analyze the influence of socio-demographic characteristics on an infant's outcome and on parent ASQ reports. Our study emphasizes the role of parental socio-economic status on neurodevelopmental outcome, as previously described [22]. Thus, the median DQ value was significantly higher among the subpopulation of infants with high socio-economic level, even though it appears difficult to conclude that this difference is clinically relevant. Regarding the status of ASQ as a screening tool, it was of prime importance to demonstrate that the social background of parents did not modify their report of their child's neurological assessment. We found no evidence that the accuracy of the ASQ reports was influenced by socio-economic level or maternal education. Our results emphasize an earlier study by ASQ authors who showed in a population of 98 parents that both middle and low income parents appeared able to complete developmental questionnaires with reasonable accuracy [23]. A previous report by Johnson et al. also provided good validity for the PRC (Parent Report Composite) irrespective of socio-demographic factors [6], whereas Heiser et al. showed a correlation between incomplete answers of the Revised Prescreening Developmental Questionnaire and a lower education level [4].

One limitation of the study is that the psychometric properties of the ASQ French version have not been studied in a control population. Nevertheless, Kerstjens et al. have demonstrated the good psychometric properties of the Dutch 48-month ASQ and the very small differences when compared to other countries [24]. Regarding a Norwegian translation, domain scores on the ASQ were similar in comparison with data from the United States [12]. Taken together, these two studies support the cross-cultural validity of the ASQ for other European countries. Our study is conducted for the first time in a French-speaking ex-premature population. The validation of the ASQ in another cultural context increases its value for international studies.

As the neurodevelopmental follow-up of ex-premature infants is costly and time-consuming, ASQ provides an interesting approach that allows the clinician to be assured of normal developmental progression in almost all infants at 2 years of age who pass the questionnaire. Thus, this simple screening instrument within the high-risk population of premature infants represents an important way to identify those infants for whom routine developmental surveillance could be less intensive. It is of prime importance, both for multidisciplinary teams and families, that ASQ be used to alleviate the burden when a child is developing normally at the age of 24 months.

The present study is also encouraging in confirming the ability of parents to assess their infant's development, as previously described [3]–[6]. The explicit use of ASQ in the assessment process has the advantage of providing parents with the opportunity of being active participants, thus reinforcing their central role as active partners to monitor the development of their infant.

We conclude that ASQ is an easy and reliable tool to predict normal neurologic outcome at 2 years in ex-premature infants. We thus believe that ASQ may be beneficial with a low-cost impact to some follow-up programs, and helps to establish a genuine sense of parental involvement.

## References

[1] Fily A, Pierrat V, Delporte V, Breart G, Truffert P (2006). Factors associated with neurodevelopmental outcome at 2 years after very preterm birth: the population-based Nord-Pas-de-Calais EPIPAGE cohort.. Pediatrics.

[2] (2006). Identifying infants and young children with developmental disorders in the medical home: an algorithm for developmental surveillance and screening.. Pediatrics.

[3] Glascoe FP, Dworkin PH (1995). The role of parents in the detection of developmental and behavioral problems.. Pediatrics.

[4] Heiser A, Curcin O, Luhr C, Grimmer I, Metze B (2000). Parental and professional agreement in developmental assessment of very-low-birthweight and term infants.. Dev Med Child Neurol.

[5] Bortolus R, Parazzini F, Trevisanuto D, Cipriani S, Ferrarese P (2002). Developmental assessment of preterm and term children at 18 months: reproducibility and validity of a postal questionnaire to parents.. Acta Paediatr.

[6] Johnson S, Marlow N, Wolke D, Davidson L, Marston L (2004). Validation of a parent report measure of cognitive development in very preterm infants.. Dev Med Child Neurol.

[7] Doig KB, Macias MM, Saylor CF, Craver JR, Ingram PE (1999). The Child Development Inventory: A developmental outcome measure for follow-up of the high-risk infant.. J Pediatr.

[8] Liao HF, Wang TM, Yao G, Lee WT (2005). Concurrent validity of the Comprehensive Developmental Inventory for Infants and Toddlers with the Bayley Scales of Infant Development-II in preterm infants.. J Formos Med Assoc.

[9] Vincer MJ, Cake H, Graven M, Dodds L, McHugh S (2005). A population-based study to determine the performance of the Cognitive Adaptive Test/Clinical Linguistic and Auditory Milestone Scale to Predict the Mental Developmental Index at 18 Months on the Bayley Scales of Infant Development-II in very preterm infants.. Pediatrics.

[10] Johnson S, Wolke D, Marlow N (2008). Developmental assessment of preterm infants at 2 years: validity of parent reports.. Dev Med Child Neurol.

[11] Squires J, Bricker D, Potter L (1997). Revision of a parent-completed development screening tool: Ages and Stages Questionnaires.. J Pediatr Psychol.

[12] Janson H, Squires J (2004). Parent-completed developmental screening in a Norwegian population sample: a comparison with US normative data.. Acta Paediatr.

[13] Skellern CY, Rogers Y, O'Callaghan MJ (2001). A parent-completed developmental questionnaire: follow up of ex-premature infants.. J Paediatr Child Health.

[14] Klamer A, Lando A, Pinborg A, Greisen G (2005). Ages and Stages Questionnaire used to measure cognitive deficit in children born extremely preterm.. Acta Paediatr.

[15] Marks K, Hix-Small H, Clark K, Newman J (2009). Lowering developmental screening thresholds and raising quality improvement for preterm children.. Pediatrics.

[16] Wood NS, Costeloe K, Gibson AT, Hennessy EM, Marlow N (2005). The EPICure study: associations and antecedents of neurological and developmental disability at 30 months of age following extremely preterm birth.. Arch Dis Child Fetal Neonatal Ed.

[17] Gottfried AW, Guerin D, Spencer JE, Meyer C (1984). Validity of Minnesota Child Development Inventory in screening young children's developmental status.. J Pediatr Psychol.

[18] Roze JC, Bureau-Rouger V, Beucher A, Branger B, Bouderlique C (2007). [Follow-up network for newborns at risk for handicap in a French region].. Arch Pediatr.

[19] Fouron JC, Gosselin J, Amiel-Tison C, Infante-Rivard C, Fouron C (2001). Correlation between prenatal velocity waveforms in the aortic isthmus and neurodevelopmental outcome between the ages of 2 and 4 years.. Am J Obstet Gynecol.

[20] Josse AR, Tang JE, Tarnopolsky MA, Phillips SM Body composition and strength changes in women with milk and resistance exercise.. Med Sci Sports Exerc.

[21] Sices L, Stancin T, Kirchner L, Bauchner H (2009). PEDS and ASQ developmental screening tests may not identify the same children.. Pediatrics.

[22] Delobel-Ayoub M, Kaminski M, Marret S, Burguet A, Marchand L (2006). Behavioral outcome at 3 years of age in very preterm infants: the EPIPAGE study.. Pediatrics.

[23] Squires J, Potter L, Bricker D, Lamorey S (1998). Parent-completed developmental questionnaires: Effectiveness with low and middle income parents.. Early Childhood Research Quarterly.

[24] Kerstjens JM, Bos AF, ten Vergert EM, de Meer G, Butcher PR (2009). Support for the global feasibility of the Ages and Stages Questionnaire as developmental screener.. Early Hum Dev.

